# Establishment of a canine model of cardiac memory using endocardial pacing via internal jugular vein

**DOI:** 10.1186/1471-2261-10-30

**Published:** 2010-06-22

**Authors:** Li Yue-Chun, Ge Li-Sha, Guang Xue-Qiang, Chen Peng, Wu Lian-Pin, Yang Peng-Lin, Tang Ji-Fei, Lin Jia-Feng

**Affiliations:** 1 Department of Cardiology, Second Affiliated Hospital of Wenzhou Medical College, Wenzhou, Zhejiang 325000, China; 2 Department of Pediatrics, Second Affiliated Hospital of Wenzhou Medical College, Wenzhou, Zhejiang 325000, China

## Abstract

**Background:**

Development of experimental animal models has played an important role in understanding the mechanisms of cardiac memory. The purpose of this study was to evaluate a new canine model of cardiac memory using endocardial ventricular pacing via internal jugular vein.

**Methods:**

Twelve Beagle dogs underwent placement of a permanent ventricular pacemaker mimicking the use of pacemakers in humans and induction of cardiac memory by endocardial ventricular pacing.

**Results:**

Cardiac memory was achieved in 11 of 12 attempts overall. Procedural mortality due to cardiac tamponade (n = 1) occurred in the first attempt. The T-wave memory persisted for 96 ± 17 minutes and 31 ± 6 days in the short-term and long-term cardiac memory groups, respectively. There were no significant differences in the heart rate, blood pressure and echocardiographic parameters in the animals between before and after ventricular pacing in the short-term and long-term cardiac memory groups. No significant pathologic changes with the light microscopy were found in the present study in all dogs.

**Conclusion:**

The model does require surgery but is not as invasive as an open-chest model. This canine model can serve as a useful tool for studying mechanisms of cardiac memory.

## Background

"Cardiac memory" (CM) is characterized by persistent but reversible T-wave inversion related to abnormal activation of the ventricle such as ventricular pacing, transient left bundle branch block, ventricular preexcitation, and ventricular tachycardia[1-4]. Ventricular pacing alters the activation sequence of ventricular depolarization, which in turn alters the repolarization sequence. The repolarization change is manifested electrocardiographically by T wave change. Following the return to sinus rhythm after an interval of abnormal ventricular depolarization, the T wave vector persists in tracking the vector angle and amplitude of the QRS complex that characterized the paced state. The T-wave memory may persist for minutes to months after either short or long periods of ventricular pacing and will be described as short-term or long-term CM, respectively. However, the exact time period required to separate short- and long-term CM is still unknown.

CM has clinical impact in that its ST-T- wave changes may mimic those of coronary ischemia, and it may modify antiarrhythmic drug efficacy and expression of rhythm and arrhythmia [5]. In addition, many studies[6-8] have demonstrated that right ventricular septal or outflow tract pacing is superior to right ventricular apical pacing, and there is a significant increase in cardiac output with septal pacing or outflow tract pacing but not with apical pacing. These studies provide strong evidence that the site of ventricular pacing has important effects on cardiac function. As the site of ventricular pacing has also been noted to affect the direction of repolarization changes that occur with CM, there may be a link between these phenomena [9]. Therefore, the clinical importance and implications of CM require us to develop a CM model.

Most previously described CM models have been surgically created via thoracotomy using epicardial pacing[4,10-12]. We have developed a closed-chest canine CM model based on permanent transvenous ventricular pacemaker implantation using right ventricular endocardial pacing.

## Methods

### Experimental animals

Eighteen healthy adult Beagle dogs (5 females and 13 males, weighing 13-15 kg) were obtained from the Experimental Animal Center of the Wenzhou Medical College. The animals were randomized into 3 groups: n = 6 in the short-term CM group; n = 6 in the long-term CM group; n = 6 in the control (sham-operated) group. The six-limb-lead electrocardiogram (ECG) was recorded and transthoracic echocardiography was examined in all dogs prior to pacemaker insertion.

### Anesthesia and pacemaker implantation

**Figure 1:**
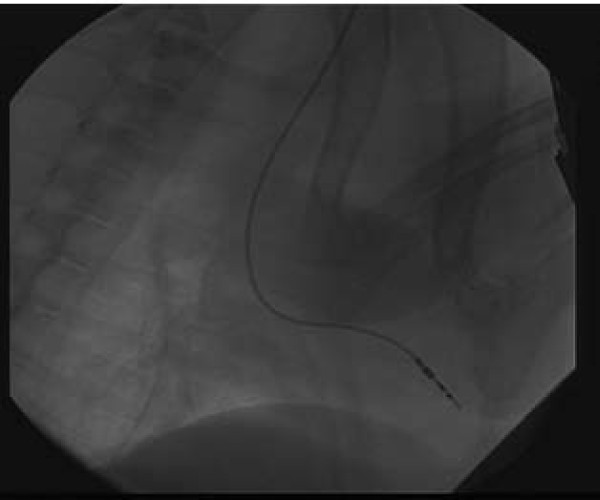
**Pacemaker lead positioned at right ventricular apex for preparing cardiac memory canine model**.

All dogs were anesthetized with an intraperitoneal injection of 3% sodium pentobarbital (30 mg/kg). Arterial pressure was continuously monitored via a catheter in the right femoral artery through a small incision in the vessel wall. Five percent glucose in normal saline (500 mL) with penicillin (4.8 million IU) was administered intravenously. The neck was prepared and draped in standard sterile surgical fashion. A small incision was made, and the right internal jugular vein was identified and dissected free of connecting tissue. Under fluoroscopy, an endocardial pacemaker lead (Medtronic, Minneapolis, USA) was inserted into the right ventricular apex from the right internal jugular vein (Fig. 1). An electronic pacemaker generator (Medtronic, Minneapolis, USA) was implanted in a small subcutaneous pocket created between the scapulas. The pacemaker lead was connected to the generator through a subcutaneous canal, and the back and neck incisions were sutured closed. Ventricular pacing was instituted (mode VVO; amplitude, 3.3 to 5 V; pulse width,0.35 to 0.5 ms) at a rate 15% faster than that of the animal's sinus rhythm, so as to ensure that the heart beats were indeed ventricularly paced. Dogs in the control group underwent a sham surgery in which the pacemaker leads and generators were installed, but not connected. The appetite, behaviour, and activity status were recorded postoperatively in all dogs.

### Induction of short-term CM and ECG Recordings

The short-term CM group was paced for one hour. After pacing was stopped, the lasting time of T-wave memory was obtained. The six-limb-lead ECG was recorded before and at the end of the ventricular pacing and every 20 minutes after the pacemaker was shut off until the short-term CM disappeared.

### Induction of long-term CM and ECG Recordings

The long-term CM group was paced for one week. After pacing was stopped, the lasting time of T-wave memory was obtained. The six-limb-lead ECG was recorded before and at the end of the ventricular pacing and every day after the pacemaker was shut off until the long-term CM disappeared.

### Echocardiographic examination

The echocardiography was performed before pacemaker implantation and after pacing was stopped in all groups and obtained the data in left ventricular ejection fraction (LVEF), ventricular chamber dimensions and wall thicknesses.

### Pathological examination of the heart

After CM disappeared, the heart was removed, heart weight and heart weight to body weight ratio was calculated. Fresh left and right ventricular tissues were obtained from each heart and fixed for 12--24 h in a 10% formalin solution. The tissues were then embedded in paraffin and sectioned. The sections were observed under optical microscope using haematoxylin and eosin (HE) staining.

### Statistical analysis

All values were expressed as mean value ± standard deviation. Student's t-test was used to compare various hemodynamic and echocardiographic parameters at baseline and at completion of model preparation. A value of P < 0.05 was considered significant.

### Ethics

The study was approved by the Wenzhou Medical University Committee on Ethics in the Care and Use of Laboratory Animals. All animals received humane care in compliance with the Guide for the Care and Use of Laboratory Animals, published by the National Institutes of Health (NIH publication No.85-23, revised 1985).

## Results

### Feasibility

11 of 12 dogs in the short-term CM and long-term CM groups successfully developed CM after ventricular pacing. One failure occurred in the first attempt, with 100% success in the latter 11 preparations in the series. The dog in the short-term CM died during pacemaker implantation and was attributed to cardiac perforation and tamponade. No CM phenomenon were seen in the control group.

The appetite, behaviour, and activity status in all dogs did not change significantly over the course of the study. There were no significant differences in the heart rate and blood pressure in the animals between before and after ventricular pacing in the short-term CM and long-term CM groups (Table 1).

**Table 1:** Hemodynamic and echocardiographic parameters at baseline and after induction of CM

	Control			Short-term CM			Long-term CM		
	
	Baseline	Postoperation	*p*-value	Baselin	Post CM	*p*-value	Baselin	Post CM	*p*-value
HR (bpm)	125 ± 15	128 ± 17	NS	122 ± 14	127 ± 15	NS	120 ± 19	124 ± 21	NS
SBP(mm Hg)	145 ± 25	139 ± 30	NS	137 ± 29	131 ± 25	NS	148 ± 35	140 ± 30	NS
DBP(mm Hg)	105 ± 16	101 ± 13	NS	99 ± 15	94 ± 13	NS	110 ± 16	103 ± 17	NS
Echocardiographic parameters									
IVSt(mm)	8.2 ± 1.0	8.3 ± 1.2	NS	9.0 ± 1.3	9.1 ± 1.5	NS	8.8 ± 0.9	8.8 ± 1.1	NS
LVPWt(mm)	7.3 ± 1.3	7.4 ± 1.5	NS	8.0 ± 1.1	8.1 ± 1.4	NS	7.6 ± 1.5	7.8 ± 1.4	NS
LVEDd (mm)	36.5 ± 5.5	38.4 ± 5.3	NS	38.1 ± 6.0	38.8 ± 6.2	NS	38.0 ± 5.6	38.2 ± 5.6	NS
RVd (mm)	16.3 ± 3.4	17.1 ± 3.5	NS	16.9 ± 3.1	17.1 ± 3.3	NS	16.5 ± 3.1	16.6 ± 3.3	NS
LVEF (%)	59.5 ± 8.6	58.0 ± 8.5	NS	61.0 ± 9.0	62.8 ± 9.2	NS	62.1 ± 9.5	60.7 ± 8.9	NS

HR, heart rate; SBP, systolic blood pressure; DBP, diastolic blood pressure; NS, not significant; IVSt, interventricular septum thickness; LVPWt, left ventricular posterior wall thickness; LVEDd, left ventricular end-diastolic diameter; RVd, right ventricular diameter; LVEF, left ventricular ejection fraction; CM, cardiac memory.

### ECG findings

**Figure 2:**
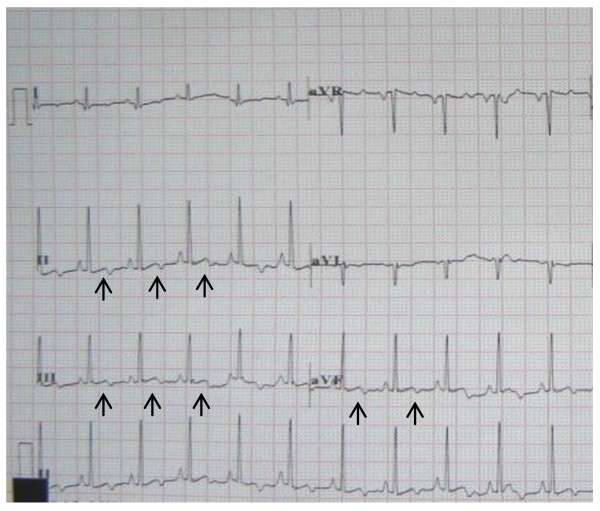
**Canine ECG with upright or biphasic T waves in leads II, III, aVF during sinus rhythm before pacing was initiated**.

**Figure 3:**
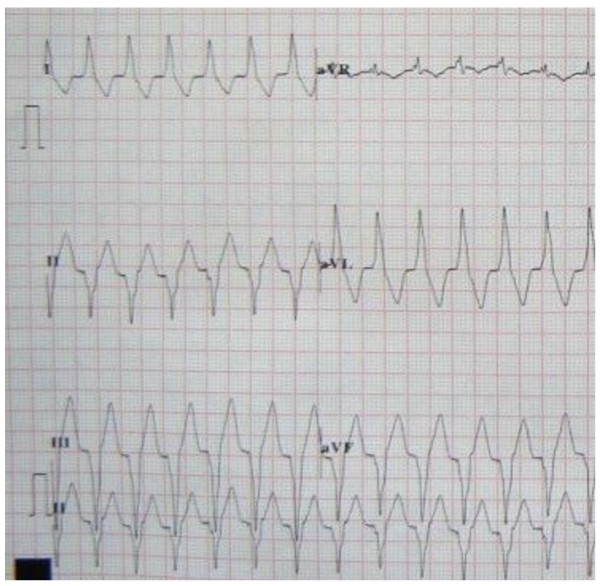
**Canine ECG during ventricular pacing**.

**Figure 4:**
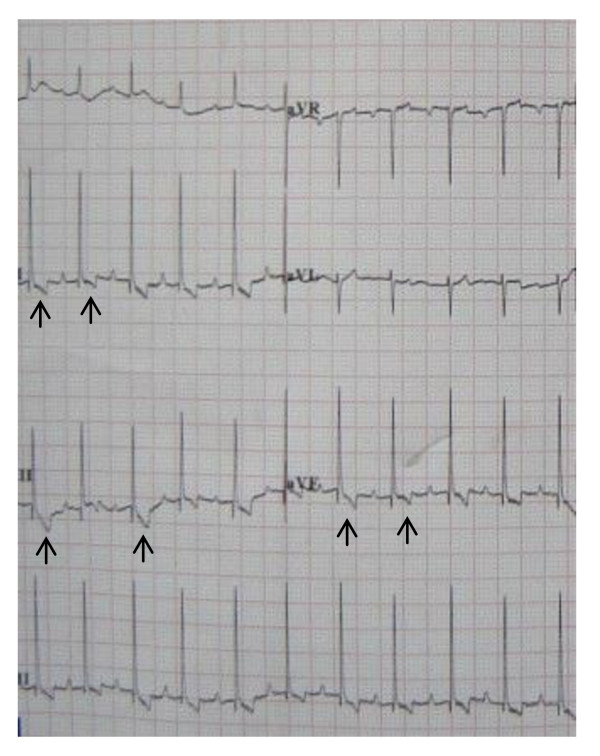
**Canine ECG during sinus rhythm with T waves inversion in leads II, III, aVF one hour after ventricular pacing in the short-term CM group**.

**Figure 5:**
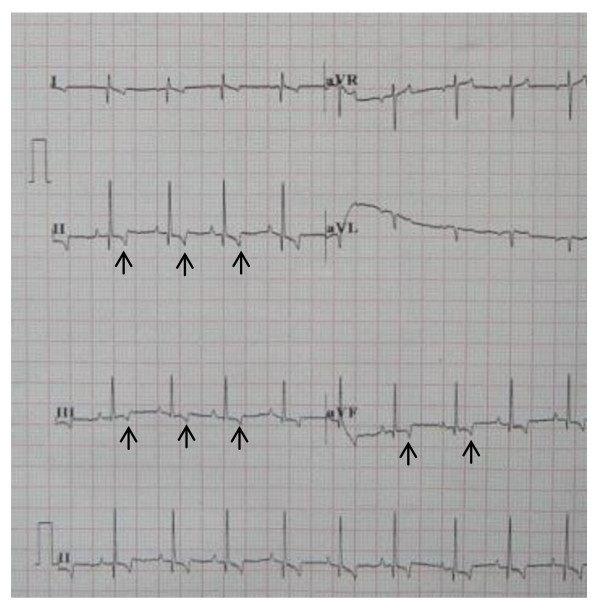
**Canine ECG during sinus rhythm with deep T waves inversion in leads II, III, aVF one week after ventricular pacing in the long-term CM group**.

All ECGs showed sinus rhythm with upright or biphasic T waves in leads II, III, aVF before pacing was initiated (Fig 2). During right ventricular apex pacing, ECGs showed wide QRS complex with upright T waves (Fig 3). In the short-term CM group, CM was successfully induced in all 5 survivors immediately after the off-set of pacing. The ECGs showed T waves inversion in leads II, III, aVF during sinus rhythm after ventricular pacing was stopped (Fig 4). The T-wave memory persisted for 96 ± 17 minutes. In the long-term CM group, CM was successfully induced in all 6 dogs after the pacemaker was turned off. The ECGs during sinus rhythm after ventricular pacing was stopped showed deep T waves inversion in leads II, III, aVF (Fig 5). The T-wave memory persisted for 31 ± 6 days.

### Echocardiographic findings

None of the echocardiographic parameters (including LVEF, right and left ventricular chamber dimensions, interventricular septum and left ventricular posterior wall thicknesses) changed significantly between before and after ventricular pacing in all dogs (Table 2).

**Table 2:** HW/BW ratio after induction of CM and lasting time of T-wave memory

Group	n	HW/BW ratio	lasting time of T-wave memory
Control	6	0.88 ± 0.11	ND
Short-term CM	5	0.90 ± 0.12	96 ± 17 minutes
Long-term CM	6	0.86 ± 0.10	31 ± 6 days

HW/BW ratio, heart weight/body weight ratio; ND, not detected.

### Pathological examination of the heart

**Figure 6:**
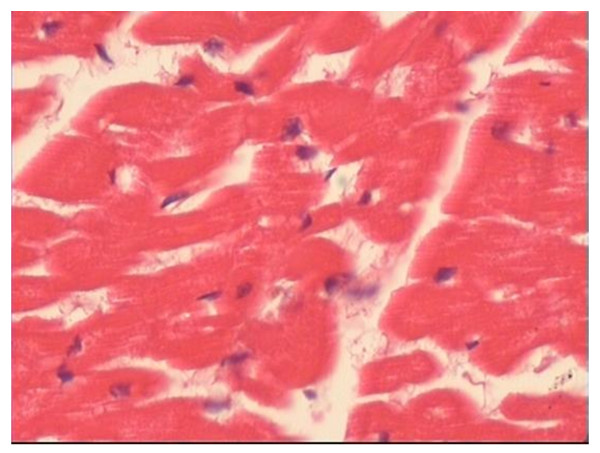
**Representative histopathology in the short-term CM group (HE × 200)**.

**Figure 7:**
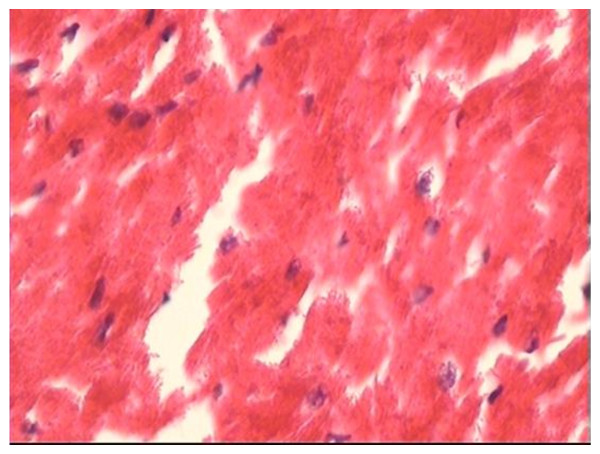
**Representative histopathology in the long-term CM group (HE × 200)**.

No differences in heart weight to body weight ratio and myocardial hispathology were found after ventricular pacing among the three groups (Table 2, Fig 6, Fig 7). On light microscopy, myofibrillar disarray, inflammatory cell infiltration and myocardial necrosis were not found in all dogs.

## Discussion and Conclusion

Although the term CM was coined by Rosenbaum in 1981 [1], the electrophysiological basis for T-wave memory and the mechanisms responsible for triggering CM have remained elusive. Identifying these mechanisms has important implications for the pathophysiology of common heart diseases involving CM [5]. Guided by these considerations, we designed the non-thoracotomy CM model in which CM was precipitated by using endocardial pacing via internal jugular vein.

CM models can provide valuable insights into several areas. One is for use in analyzing mechanisms of CM. Another is that it offers the possibility of evaluating novel strategies for targeted prevention of CM. CM models can also be used to assess the effects of short-term and long-term CM on cardiac function. Recognition of these advantages has led to development of several CM models. Shvilkin et al. [4] described a method inducing CM using epicardial pacing via thoracotomy in mongrel dogs. Approaching the heart through a thoracotomy at the fifth left intercostal space, epicardial pacing lead was attached to the inferolateral left ventricular wall. Then the lead was connected to a dual-chamber pacemaker affixed subcutaneously. The animal model of CM was frequently used in recent years[10-12]. Some studies[10,13] using the model have demonstrated that the transient outward potassium current (*I*_to_), epicardial mRNA levels of Kv4.3 (one of the molecular correlates of *I*_to_) and L-type Ca^2+ ^channel (*I*_Ca,L_) may be involved in the development and maintenance of cardiac memory. Nevertheless, the method inducing CM via thoracotomy had severe surgical trauma, may result in increasing dog mortality.

In the present study, our model minimized surgical trauma using endocardial pacing via internal jugular vein. In our model, just one dog died during the early part of the experience. Avoidance of thoracotomy should ultimately enhance survival. Moreover, we mimicked the use of pacemakers in humans. The method to induce CM in our model appears more similar to clinical situations in humans. Therefore, it may have an application in analyzing mechanisms of CM.

This canine model had a high success rate (11/12, 91.7%). After successful pacemaker implantation, all dogs in short-term and long-term CM groups develop the CM model. In our experience, the key points to develop the CM animal model are as follows: the operation of pacemaker implantation should be under fluoroscopy; right ventricular pacemaker lead insertion via internal jugular vein should be gentle, otherwise maybe result in cardiac perforation and tamponade.

In agreement with some previous studies [4,10-12], we found that the appetite, behaviour, and activity status in all dogs did not change significantly over the course of the study, and there were no significant differences in the heart rate, blood pressure and echocardiographic parameters in the animals between before and after ventricular pacing in the short-term CM and long-term CM groups. Moreover, we demonstrated no significant pathologic changes with the light microscopy in the present study. The result was similar to that of previously reported studies [14,15]. In addition, these studies [14,15] also found that right ventricular pacing was associated with mismatching of perfusion and innervation with perfusion abnormalities of both the septum and free wall. However, other studies [16,17] have identified ultrastructural changes induced by pacing. Karpawich et al. [16] found that after the dogs with epicardial pacemaker lead placed near the right ventricular apex through a thoracotomy were paced for several months, myofibrillar disarray, and areas of dystrophic calcification dispersed throughout the left ventricular free wall on light microscopy. It is unclear whether the different findings by us reflect different pacing modes or pacing time or surgical method or other technical differences between our study and that of Karpawich et al.

In conclusion, a method of establishing a canine model of CM using endocardial pacing via internal jugular vein was successfully demonstrated and it proved to be simple, safe and effective. The ECGs characteristics of the model are in good accordance with those of CM in patients. The novel CM model described here can offer potentially important advantages over preexisting models.

## Competing interests

The authors declare that they have no competing interests.

## Authors' contributions

LJF and LYC designed the whole study, LYC, GLS, GXQ, CP, WLP, YPL, and TJF performed the experiment, LYC and GLS wrote the paper. All authors read and approved the final manuscript.

## Pre-publication history

The pre-publication history for this paper can be accessed here:

http://www.biomedcentral.com/1471-2261/10/30/prepub
